# Prevalence of Spaced and Closed Dentition and its Relation to Malocclusion in Primary and Permanent Dentition

**DOI:** 10.5005/jp-journals-10005-1144

**Published:** 2012-08-08

**Authors:** Suma Vinay, Vinayk Keshav, Shreya Sankalecha

**Affiliations:** Reader, Department of Pedodontics and Preventive Dentistry VS Dental College and Hospital, Bengaluru, Karnataka, India, e-mail: sumadoc@yahoo.co.in; Reader, Department of Orthodontics and Dentofacial Orthopedics, MR Ambedkar Dental College and Hospital, Bengaluru, Karnataka, India; Postgraduate Student, Department of Pedodontics and Preventive Dentistry, VS Dental College and Hospital, Bengaluru, Karanataka, India

**Keywords:** Spacing, Closed dentition and malocclusion

## Abstract

An ideal primary dentition is the indicator of future ideal permanent dentition. Absence of primate or secondary spaces in the primary dentition is expression of disproportion between jaw/tooth sizes. Little information is known on the relationship of spacing and closed dentition with malocclusion in relation to primary and permanent dentition. Hence, the present study was conducted to find the relationship of spacing and closed dentition with malocclusion in primary and permanent dentition in children during their deciduous dentition period.

## INTRODUCTION

Childhood is a mirror in which, the propensity of adulthood is reflected. Similarly an ideal primary dentition is the indicator of future ideal permanent dentition.^1^ It is very common to find physiologic spaces in primary dentition. The prevalence of spaced dentition varies between different ethnic groups ranging from 42 (Triemann 1961) to 98% (Byoke 1968).^2^

These spaces are very important in the later stage for alignment of erupting permanent teeth and establishment of occlusion. Absence of these spaces in the primary dentition is an expression of disproportion between jaw/tooth sizes. The establishment and maintenance of normal occlusion constitute one of the important objectives of pedodontic treatment whether it is preventive, interceptive or corrective.^3^ As little information is known on the state of occlusion and the space available at the time of completion of eruption of primary dentition, this study was done in order to find out the relationship of spacing and closed dentition with malocclusion in relation to primary and permanent dentition, in Bengaluru children below 6 years of age.

## AIMS AND OBJECTIVES

The aim of this study is to know the prevalence of spaced and closed primary dentition by sex in schoolchildren in Bengaluru during their deciduous dentition period.

## MATERIALS AND METHODS

One thousand schoolchildren below 6 years of age having all deciduous teeth were examined for spacing and closed dentition in relation to primary dentition.

### Inclusion Criteria

1. The schoolchildren below 6 years of age.
2. All deciduous teeth should be present.
3. Presence of spacing and closed dentition is assessed.
4. Other abnormalities like presence of nonvital teeth supernumerary/ supplemental teeth, fusion, missing teeth, submerged teeth, rampant caries and nursing bottle caries.

### Exclusion Criteria

Eruption of any permanent first molar/incisor tooth–not taken as subject.

## RESULTS

**Graph 1:**
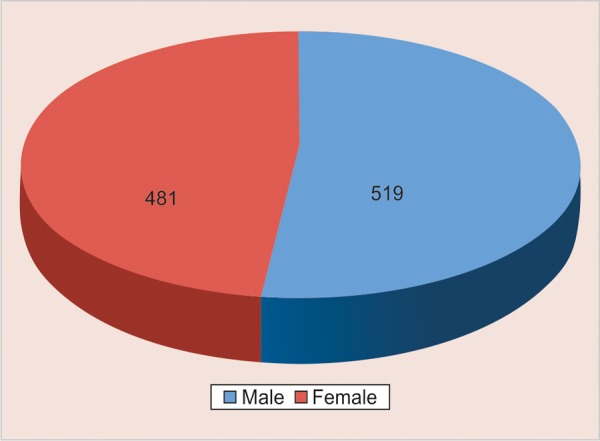
Distribution of sample according to sex

**Graph 2:**
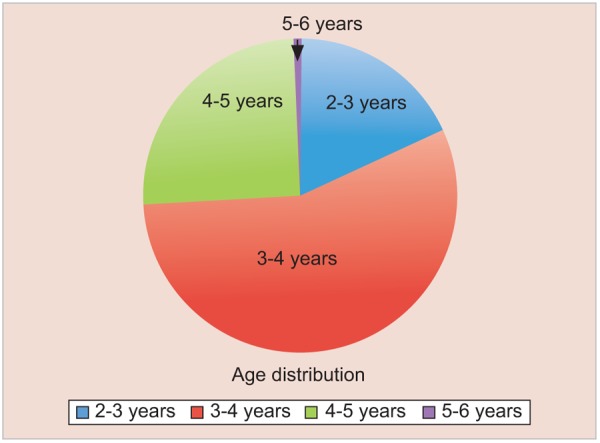
Age distribution of 1000 children

**Graph 3:**
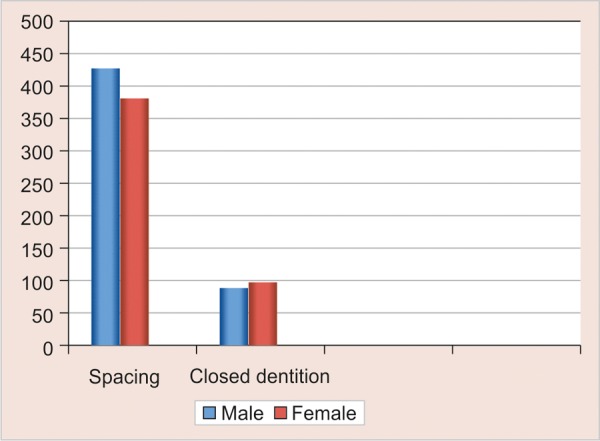
Distribution of alignment

1. *Sample size*: A total number of thousand schoolchildren below 6 years of age were taken for the study to find out the relationship of spacing and closed dentition with malocclusion in primary dentition.
  Out of thousand children, 51.9% of them were male and 48.1% of them were female children (Table 1 and Graph 1).
2. *Age distribution*: The age distribution of the thousand schoolchildren is as follows (Table 2 and Graph 2).
  - 18.1% (181) were of 2 to 3 years
  - 56.1% (561) were 3 to 4 years
  - 25.2% (252) were 4 to 5 years
  - 0.06% (06) were of 5 to 6 years.
3. *Alignment*: Eighty-one percent of children had spaced dentition and 19% of children had closed dentition respectively. The distribution o f alignment is shown in Table 3 and Graph 3.
4. *Other abnormalities*: Very few children in the study group had abnormalities like fusion of teeth (0.2%), enamel hypoplasia (0.2%) and one case each of scissor bite, rampant caries and fluorosis.

## DISCUSSION

The presence of spaced or closed dentition in the primary dentition and its significance for the development of permanent dentition has long been a subject of discussion. There have been many studies of the primary dentition in preschool children in several ethnic groups.^4,5^ However, the ages of children examined ranged from 2.5 to 5 years. Bishara et al reported that the maxillary and mandibular intercanine and intermolar widths significantly increase between 3 and 5 years of age. Therefore, the subjects in this study were limited to those below 6 years of age.^8^

**Table 1:** **Table 1:** Distribution of sample according to sex

*Males*		*Females*		*Total*	
519 (51.9%)		481 (48.1%)		1000	

**Table 2:** **Table 2:** Age distribution of 1000 children

*No.*		*Age*		*Males*		*Females*		*Total*		*Percentage*	
1		2-3		95		86		181		18.1	
2		3-4		277		284		561		56.1	
3		4-5		142		110		252		25.2	
4		5-6		04		02		06		0.6	

Joshi and Makhija studied the primary dentition of 100 children, 3 to 6 years from Gujarat and reported that spaced dentition was more common than the closed type.^6^ They found that amount of spacing was greater in males, which was similar to our study where 81% had spaced dentition. Otuyemi et al examined 525, 3 to 4 years old Nigerian children and observed that 32% had generalized anterior segment spacing, 4% showed exclusively primate space and 18% had either contact between all teeth or anterior crowding.^7^ Alexander and Prabu reported that 75% of south Indians had both physiologic and primate spaces in both arches.^1^ Absence of primate or secondary spaces in the primary dentition is expression of disproportion between jaw/tooth sizes.

**Table 3:** **Table 3:** Distribution of alignment

*Spacing*						*Closed dentition*					
*Males*		*Females*		*Total*		*Males*		*Females*		*Total*	
429		381		810 (81%)		89		101		190 (19%)	

Dong-Hyuk Im et al showed that in both sexes spacing in the primary dentition was more frequent in the maxilla than in the mandible.^9^ Baume reported that there was either contact between the teeth or crowding in the mandible in 14.9% of children.^10^ El-Nofely et al reported that children with spaced dentition have small mesiodistal crown diameter and wide inter-canine width.^11^ Leighton's hypothesis suggests that there should be 6 mm or more space between the mandibular teeth in order for there to be no chance of developing incisor crowding in the permanent dentition. Hence, the results in our present study showed 81% children had spacing who have less chances of developing malocclusion in permanent dentition, 19% of children had closed dentition which showed that these children have more frequency of developing malocclusion and our study also showed that spaced dentition was seen more in males when compared to females depicting that the frequency of developing malocclusion was more in females than in males.

## CONCLUSION

The study provides the information on prevalence of spacing and closed dentition in the primary dentitions of Bengaluru urban children. Spaced dentition was more frequent than closed dentitions. Spaced dentition is seen more in males compared to female children. Closed dentition is seen more in female children than males depicting that the frequency of developing malocclusion in permanent dentition was more in females than in males.
